# The use of gabapentin in the management of postoperative pain after total hip arthroplasty: a meta-analysis of randomised controlled trials

**DOI:** 10.1186/s13018-016-0412-z

**Published:** 2016-07-12

**Authors:** Chao Han, Xiao-dan Li, Hong-qiang Jiang, Jian-xiong Ma, Xin-long Ma

**Affiliations:** Department of Orthopedics, Tianjin Hospital, No. 406 Jiefang South Road, Hexi District Tianjin City, 300211 People’s Republic of China; Department of Anesthesiology, Tianjin First Central Hospital, No.24 Fukang Road, Nankai District Tianjin City, 300192 People’s Republic of China

**Keywords:** Gabapentin, Total hip arthroplasty, Meta-analysis

## Abstract

**Background:**

Pain management after total hip arthroplasty (THA) varies and has been widely studied in recent years. Gabapentin as a third-generation antiepileptic drug that selectively affects the nociceptive process has been used for pain relief after THA. This meta-analysis was conducted to examine the efficacy of gabapentin in THA.

**Methods:**

An electronic-based search was conducted using the following databases: PubMed, EMBASE, Ovid MEDLINE, ClinicalTrials.gov, and Cochrane Central Register of Controlled Trials (CENTRAL). Randomised controlled trials (RCTs) involving gabapentin and a placebo for THA were included. The meta-analysis was performed following the Preferred Reporting Items for Systematic Reviews and Meta-Analyses (PRISMA) statement.

**Results:**

Five trials met the inclusion criteria. The cumulative narcotic consumption and the visual analogue scale (VAS) scores at 24 and 48 h postoperatively were used for postoperative pain assessment. There was a significant decrease in morphine consumption at 24 h (*P* = 0.00). Compared with the control group, the VAS score (at rest) at 48 h was less in the gabapentin group (*P* = 0.00).

**Conclusion:**

The administration of gabapentin is effective in decreasing postoperative narcotic consumption and the VAS score.

## Background

Total hip arthroplasty (THA) is a common and successful surgery in modern medicine, but it is often associated with intense postoperative pain [1]. Pre-emptive analgesia might be a good way to relieve the postoperative pain in the clinic. However, effective treatment of postoperative pain continues to be a challenge for orthopaedists because poor control of postoperative pain can have negative effects on the pulmonary system and cardiovascular system, which can influence surgical outcomes, and it has been reported that the treatment of postoperative pain often remains insufficient [2].

The management of pain after THA is often directed at the reduction of pain and reducing morphine requirements by multimodal analgesia techniques [3]. Despite the multimodal approach, some patients may develop intractable postoperative pain [4, 5]. Currently, certain doses of opioids through patient-controlled analgesia (PCA) devices are often used for postoperative analgesia after THA [6, 7]. Although not every patient needs the additional non-opioid, the use of an additional non-opioid agent is often recommended, given the various side effects of analgesic opioids [8]. One of the agents used is gabapentin, which is a third-generation antiepileptic drug that selectively affects the nociceptive process [9]. It has not only the central and peripheral antalgic activity but also the relatively well-tolerated property [10]. In addition to gabapentin, the non-steroidal anti-inflammatory drugs (NSAIDS) as the non-opioids were also used for THA. However, the adverse effects on the gastrointestinal and haematological systems as well as on the renal functions were easily found once NSAIDS combined with opioids.

In previous years, some studies were placed to estimate the effects of pre-emptive gabapentin before surgery [11–14]. Although some studies have made their own conclusions, the role of gabapentin in postoperative pain relief after THA has not been investigated through a meta-analysis. The aim of this work was to investigate the effect of the gabapentin and make a better understanding of the efficacy and safety of gabapentin in the management of postoperative pain after THA.

## Methods

**Fig. 1:**
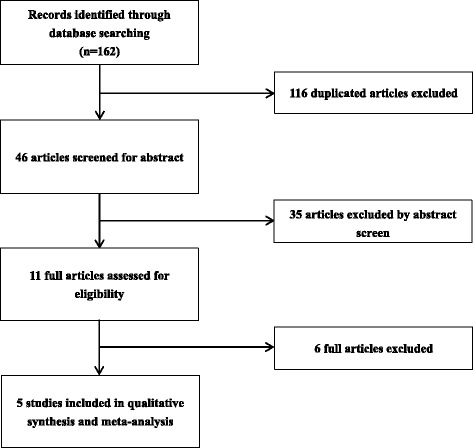
The selection of literature for included studies

This study followed the guidelines of the Preferred Reporting Items for Systematic Reviews and Meta-Analyses (PRISMA) statement [15]. We conducted an electronic-based search using the following databases: MEDLINE, PubMed, EMBASE, and Cochrane Central Register of Controlled Trials. The following medical subject heading terms, keywords, and their combinations were used: “pain management, postoperative pain, total hip arthroplasties, total hip replacement, and gabapentin”. The search was limited to randomised controlled trials (RCTs) in humans and published in English up to December 2015. The flow chart of study selection is shown in Fig. 1.

### Inclusion criteria

Studies were considered eligible for inclusion if they met the following criteria. Study design: RCTs with placebo-controlled report in English. Population: Patients with total hip arthroplasties, spinal anaesthesia only, no other local anaesthetic agent was used. Intervention group: gabapentin. Control group: placebo. Outcomes: reported at least one of the following items: postoperative consumption of morphine, pain scores (visual analogue scale (VAS)), and treatment side effects.

### Exclusive criteria

Patients were excluded from the meta-analysis if they had neoplastic aetiology, infection, traumatic fracture, metal sensitivity, or mental diseases.

### Selection criteria

An eligibility assessment was performed independently in an unblended standardised manner by two reviewers. Disagreements between reviewers were resolved by consensus. The Cochrane collaboration’s tool for the assessment of the risk of bias was used [16]. Funnel plots were drawn to assess the quality of the RCTs.

### Data extraction

Two authors (Chao Han and Hong-qiang Jiang, assigned by Xin-long Ma) independently extracted the data from the included literature. Trials were analysed and the following data were extracted: first author’s last name; publication year, gabapentin dose and regimen, type of surgery, number of patients, pain assessment methods, types and methods of administration of rescue narcotics, and adverse outcomes.

### Statistical analysis

The data were analysed by RevMan 5.3 (The Cochrane Collaboration, Oxford, UK). Heterogeneity was estimated depending on the value of *P* and *I*^2^ using the standard chi-square test. *P* <0.10 and *I*^2^ >50 % were defined as having significant heterogeneity. Then, a random-effects model was applied for data analysis. A fixed-effects model was used when no significant heterogeneity was found. The results of the meta-analysis studies were expressed as the standardised mean difference, with 95 % confidence intervals (CIs) for continuous outcomes such as narcotic consumption and pain scores, and relative risk with 95 % CIs for dichotomous data such as nausea and other side effects. Differences in means were considered significant with a *P* < 0.05.

## Results

### Literature search

A total of 162 potential studies were identified with the first search strategy, and 116 were removed as duplicates. The remainder of the 46 records was screened. After assessment of the titles and abstracts, 35 articles were excluded as irrelevant. In total, 11 potentially eligible studies were identified, six of which were excluded, leaving five studies that met the eligibility criteria [17–21]. The pooled data consisted of 269 patients in the gabapentin group and 304 patients in the control group. These five studies were published between the years of 2009 and 2015. Each study included between 20 and 300 patients. In three trials, gabapentin was given preoperatively only [18, 20, 21], whereas in the two other trials, gabapentin were administered preoperatively and postoperatively [17, 19]. “Clarke 2009 Pre” and “Clarke 2009 Post” was the same trial, in which there were three groups, and we divided this trial into two different comparisons (gabapentin vs. placebo preoperatively; gabapentin vs. placebo postoperatively). “Clarke 2010 Pre” and “Clarke 2010 Post” was also the same trial, in which there were three groups, and we divided this trial into two different comparisons (gabapentin vs. placebo preoperatively; gabapentin vs. placebo postoperatively).

### Study characteristics

The characteristics of the included gabapentin studies are reported in Table 1. Statistically similar baseline characteristics were observed between the gabapentin and placebo groups Table 1.

**Table 1:** Characteristics of included studies

Clinical trials	Age(years)	Gender (M/F)	Location	No. of patients gabapentin/control	Dose of gabapentin	Time of gabapentin administration
Clarke 2009 Pre	60.1	50/29	Canada	40/39	600 mg preoperatively	2 h preoperatively
Clarke 2009 Post	60.9	43/34	Canada	38/39	600 mg postoperatively	In the recovery room
Clarke 2010 Pre	60.4	45/28	Canada	38/38	600 mg preoperatively	2 h preoperatively
Clarke 2010 Post	61.7	44/35	Canada	38/38	600 mg postoperatively	In postanesthetic care unit
Clarke 2010	60.8	43/27	Canada	22/48	600 mg	2 h preoperatively
Nantha 2011	61.1	52/41	Canada	45/48	800 mg on day 0, and 200 mg tid for 2 days	2 h preoperatively and postoperatively
Paul 2015	60.7	58/44	Canada	48/54	600 mg	2 h preoperatively

### Risk of bias assessment

**Fig. 2:**
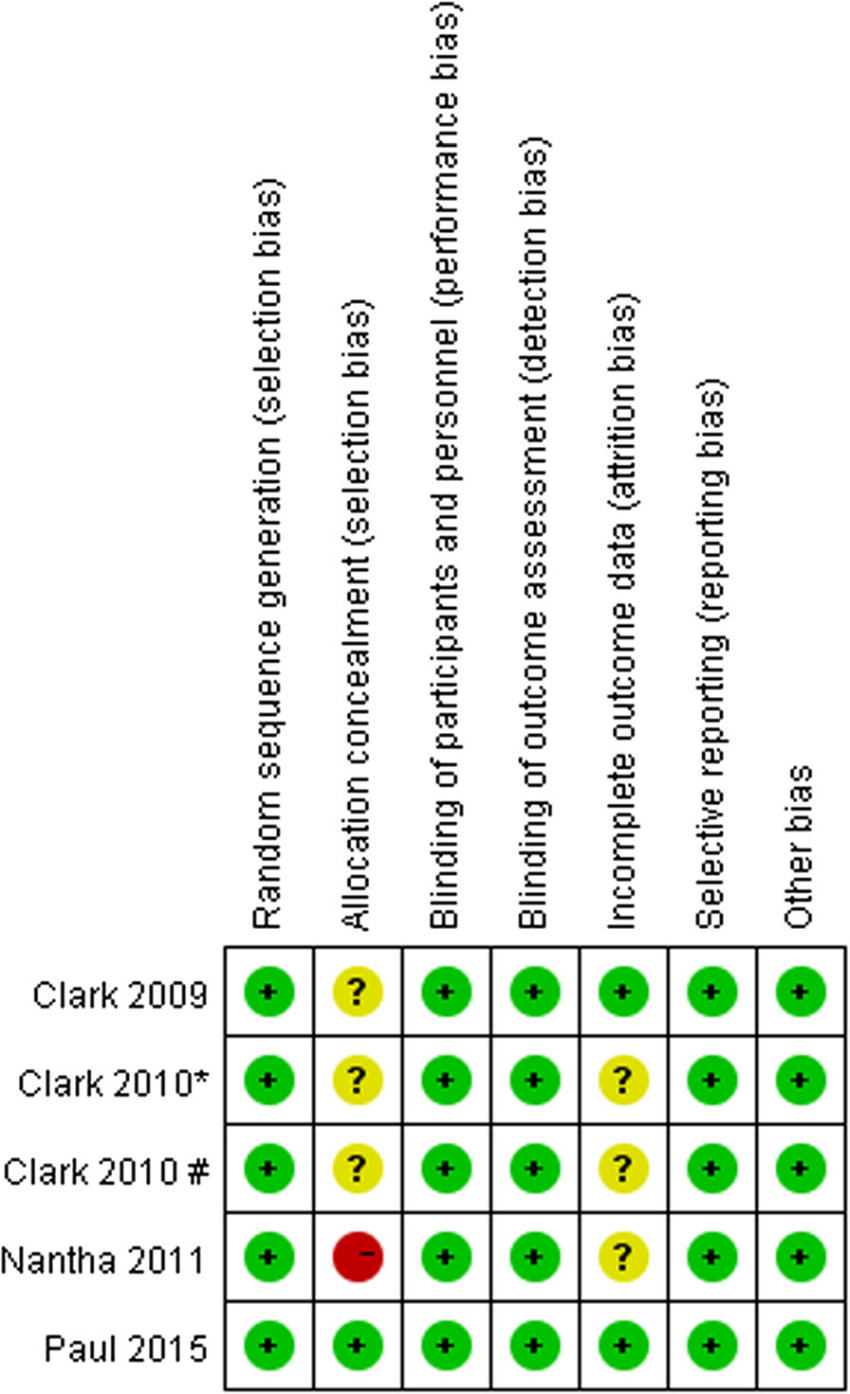
The summary of bias risk of randomised controlled trials

According to the Cochrane collaboration’s tool for assessing the risk of bias in RCTs, all our included trials have a low risk for bias Fig. 2.

### Outcomes for meta-analysis

#### Postoperative narcotic requirements at 24 h

**Fig. 3:**
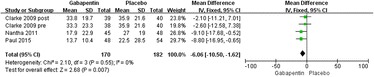
Forest plot of postoperative narcotic consumption at 24 h between the two groups

Details regarding narcotic consumption at 24 h were available in four trials [19–21]. There was no significant heterogeneity (*χ*^2^ = 2.10, *df* = 3, *I*^2^ = 0 %, *P* = 0.55); therefore, a fixed model was performed. The overall pooled results from the meta-analysis showed that compared with placebo, gabapentin could significantly reduce postoperative narcotic consumption (MD = −6.06, 95 % CI −10.50 to −1.62, *P* = 0.007; Fig. 3).

#### Postoperative narcotic requirements at 48 h

**Fig. 4:**
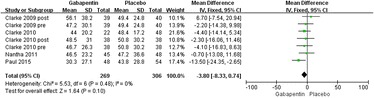
Forest plot of postoperative narcotic consumption at 48 h between the two groups

Details regarding narcotic consumption at 48 h were available in seven trials [17–21]. There was no significant heterogeneity (*χ*^2^ = 5.53, *df* = 6, *I*^2^ = 0 %, *P* = 0.48); therefore, a fixed model was performed. The overall pooled results from the meta-analysis showed that compared with placebo, gabapentin could not significantly reduce postoperative narcotic consumption (MD = 3.80, 95 % CI −8.30 to 0.70, *P* = 0.10; Fig. 4).

#### Postoperative VAS (at rest) at 24 h

**Fig. 5:**
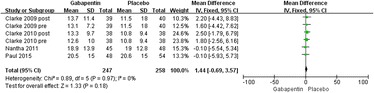
Forest plot of postoperative VAS (at rest) at 24 h between the two groups

Six trials reported VAS at 24 h [17, 19–21]. Significant heterogeneity was not found; therefore, a fixed model was used (*χ*^2^ = 0.89, *df* = 5, *I*^2^ = 0 %, *P* = 0.97). Compared with placebo, gabapentin could not significantly reduce the VAS at 24 h (MD = 1.44, 95 % CI −0.69 to 3.57, *P* = 0.18; Fig. 5).

#### Postoperative VAS (with movement) at 24 h

**Fig. 6:**
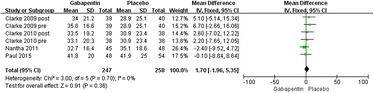
Forest plot of postoperative VAS (with movement) at 24 h between the two groups

Six trials reported VAS at 24 h [17, 19–21]. There was no significant heterogeneity (*χ*^2^ = 3.00, *df* = 5, *I*^2^ = 0 %, *P* = 0.70); therefore, a fixed model was performed. The overall pooled results from meta-analysis showed that compared with placebo, no significant difference was found in the gabapentin groups (MD = 1.70, 95 % CI −1.96 to 5.35, *P* = 0.91; Fig. 6).

#### Postoperative VAS (at rest) at 48 h

**Fig. 7:**
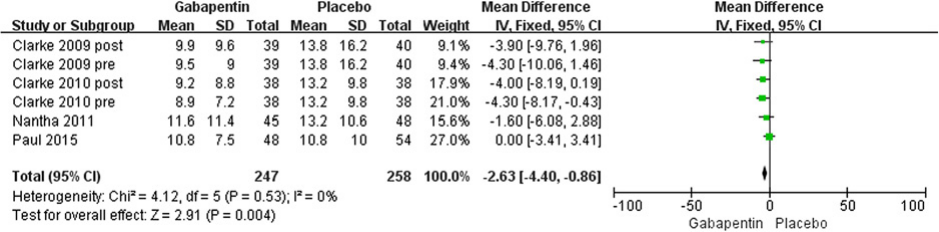
Forest plot of postoperative VAS (at rest) at 48 h between the two groups

Six trials reported VAS at 48 h [17, 19–21]. Significant heterogeneity was not found; therefore, a fixed model was used (*χ*^2^ = 4.12, *df* = 5, *I*^2^ = 0 %, *P* = 0.53). The pooled results showed that compared with placebo, gabapentin could significantly reduce the VAS at 48 h (MD = −2.63, 95 % CI −4.40 to −0.86, *P* = 0.004; Fig. 7).

#### Postoperative VAS (with movement) at 48 h

**Fig. 8:**
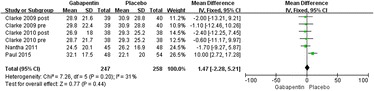
Forest plot of postoperative VAS (with movement) at 48 h between the two groups

Six trials reported VAS at 48 h [17, 19–21]. There was no significant heterogeneity (*χ*^2^ = 7.26, *df* = 5, *I*^2^ = 31 %, *P* = 0.20); therefore, a fixed model was performed. The overall pooled results from the meta-analysis showed that compared with placebo, no significant difference was found in the gabapentin groups (MD = 1.47, 95 % CI −2.28 to 5.21, *P* = 0.44; Fig. 8).

#### Adverse effects

**Fig. 9:**
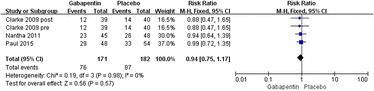
Forest plot of incidence of nausea between the two groups

The most commonly reported adverse effect in the trials included in our study was nausea, which was reported in four studies [19–21]. Significant heterogeneity was not found; therefore, a fixed model was used (*χ*^2^ = 0.19, *df* = 3, *I*^2^ = 0 %, *P* = 0.98). Compared with the control group, no significant difference was found in the gabapentin groups (relative rate 0.94, 95 % CI 0.75–1.17, *P* = 0.57; Fig. 9).

**Fig. 10:**
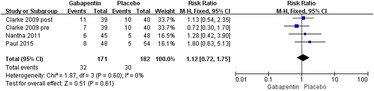
Forest plot of incidence of pruritus between the two groups

Four studies reported the incidence rate of pruritus [19–21]. Significant heterogeneity was not found; therefore, a fixed model was used (*χ*^2^ = 1.87, *df* = 3, *I*^2^ = 0 %, *P* = 0.60). Compared with the control group, no significant difference was found in the gabapentin groups (relative rate 1.12, 95 % CI 0.72–1.75, *P* = 0.61; Fig. 10).

**Fig. 11:**
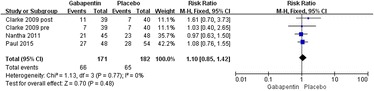
Forest plot of incidence of sedation between the two groups

Four studies reported the incidence rate of sedation [19–21]. Significant heterogeneity was not found; therefore, a fixed model was used (*χ*^2^ = 1.13, *df* = 3, *I*^2^ = 0 %, *P* = 0.77). Compared with the control group, no significant difference was found in the gabapentin groups (relative rate 1.10, 95 % CI 0.85–1.42, *P* = 0.48; Fig. 11).

**Fig. 12:**
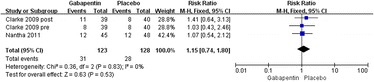
Forest plot of incidence of dizziness between the two groups

Three studies reported the incidence rate of dizziness [19, 20]. Significant heterogeneity was not found; therefore, a fixed model was applied (*χ*^2^ = 0.36, *df* = 2, *I*^2^ = 0 %, *P* = 0.83). Compared with the control group, no significant difference was found in the gabapentin groups (relative rate 1.15, 95 % CI 0.74–1.80, *P* = 0.53; Fig. 12).

## Discussion

This work aimed to review the related papers systematically to gain a better understanding of the efficacy of gabapentin in the treatment of postoperative pain after THA. Our results showed that compared with the control group, a significant reduction in cumulative narcotic consumption was found at 24 h postoperatively. This finding shares the same views with previous systematic studies examining the effect of gabapentin in different surgeries [12, 22, 23].

Another technique used to evaluate the effect of gabapentin in the treatment of pain was to assess the pain scores. In this part, the 24- and 48-h postoperative VAS score (at rest or with movement) was chosen as our point of comparison. However, a significant reduction in VAS score was only found in the movement group at 48 h postoperatively, when compared with placebo. There was no significant difference in the rest of the groups. This finding of our research is different from those of previous studies [23, 24]. This could be explained by the discrepancy of the surgical procedure and the difference of sample sizes.

Nausea is the one of adverse effects in the postoperative period. It is related to many factors, such as the different methods of anaesthesia or opioid use. As shown in Fig. 9, the rate of nausea appeared to increase in the control group, but the difference was not statistically significant. Gabapentin administration is associated with decreased postoperative nausea, which is somewhat similar with the previous research [25]. Regarding other side effects, such as pruritus, sedation, and dizziness, we found that the gabapentin group has a similar incident rate to that of the placebo group. It is still unclear whether these side effects are dose-related.

As we know it, this study might be the first meta-analysis regarding gabapentin in the management of postoperative pain after THA. To overcome the shortcomings of retrospective or observational studies, all of the included papers were randomised and prospective studies. The limitations of this study are the various study designs and the analytical approach, which may lead to the obvious heterogeneity in those studies. Other potentially limiting factors of this study include the type of THA, duration of surgery, and complications, which could also play a factor in the degree of pain experienced. Nearly all of the included studies were conducted by anaesthetists; therefore, some details such as surgical approach, way of incision, fixation method, and variety of implant were rarely reported in those studies. However, that information was usually crucial to orthopaedics. It is believed that all of these factors have the ability to change the degree of postoperative pain, so they need to be taken into account in future studies.

Another limitation was that the dosages and administration time of gabapentin were inconsistent; the dose was 600 mg in some trials and 800 mg in others. However, our analysis demonstrated that the gabapentin only had a significant effect on VAS scores in the movement group at 48 h. Pandey et al. [26] suggested that 600 mg of gabapentin was the best dose. However, Khan et al. [27] found that the pain scores were lower in groups receiving 900 or 1200 mg of gabapentin. We are unable definitely state what the optimal dosages of gabapentin are from this meta-analysis, yet it seems as if 600 mg has the same effect to 800 mg. Therefore, further studies are needed to be performed to further evaluate the best dose of gabapentin.

## Conclusions

This meta-analysis of prospective studies shows that gabapentin was efficacious in the reduction of postoperative narcotic requirements and VAS score after THA.

## Abbreviations

CI, confidence intervals; MD, mean difference; PRISMA, Preferred Reporting Items for Systematic Reviews and Meta-Analyses; RCTs, randomised controlled trials; RR, relative risk; THA, total hip arthroplasty; VAS, visual analogue scale scores
